# Distribution of tumor-infiltrating-T-lymphocytes and possible tumor-escape mechanisms avoiding immune cell attack in locally advanced adenocarcinomas of the esophagus

**DOI:** 10.1007/s12094-021-02556-2

**Published:** 2021-02-10

**Authors:** M. Schoemmel, H. Loeser, M. Kraemer, S. Wagener-Ryczek, A. Hillmer, C. Bruns, M. Thelen, W. Schröder, T. Zander, A. Lechner, R. Buettner, H. Schlösser, F. Gebauer, A. Quaas, Gastrointestinal Cancer Group Cologne (GCGC), H. Loeser, T. Zander, F. Gebauer, A. Quaas

**Affiliations:** 1 Department of General, Visceral and Cancer Surgery, University of Cologne, Cologne, Germany; 2 Institute of Pathology, University-Hospital of Cologne, University of Cologne, Kerpener Strasse 62, 50937 Cologne, Germany; 3 Department I of Internal Medicine, Center for Integrated Oncology (CIO), University of Cologne, Cologne, Germany; 4 LMU University-Hospital Head-Neck-Department, Munich, Germany

**Keywords:** Adenocarcinoma of the esophagus, Inflammation, Computer applications software, MHC1, PD-L1

## Abstract

**Introduction:**

The inflammatory microenvironment has emerged as one of the focuses of cancer research. Little is known about the immune environment in esophageal adenocarcinoma (EAC) and possible tumor-escape mechanisms to avoid immune cell attack.

**Patients and methods:**

We measured T cell inflammation (CD3, CD8) in the microenvironment using a standardized software-based evaluation algorithm considering different predefined tumor areas as well as expression of MHC class 1 and PD-L1 on 75 analyzable primarily resected and locally advanced (≥ pT2) EACs. We correlated these findings statistically with clinical data.

**Results:**

Patients with high amounts of T cell infiltration in their tumor center showed a significant survival benefit of 41.4 months compared to 16.3 months in T cell poor tumors (*p* = 0.025), although CD3 fails to serve as an independent prognostic marker in multivariate analysis. For the invasion zone, a correlation between number of T-cells and overall survival was not detectable. Loss of MHC1 protein expression on tumor cells was seen in 32% and PD-L1 expression using the combined positive score (CPS) in 21.2%. Most likely due to small numbers of cases, both markers are not prognostically relevant, even though PD-L1 expression correlates with advanced tumor stages.

**Discussion:**

Our analyses reveal an outstanding, though not statistically independent, prognostic relevance of T-cell-rich inflammation in our group of EACs, in particular driven by the tumor center. For the first time, we describe that the inner part of the invasion zone in EACs shows significantly fewer T-cells than other tumor segments and is prognostically irrelevant. We also demonstrate that the loss of antigen presenting ability via MHC1 downregulation by the carcinoma cells is a common escape mechanism in EACs. Future work will need to show whether tumors with MHC class 1 loss respond less well to immunotherapy.

## Introduction

Esophageal adenocarcinoma (EAC) is associated with the sixth-highest cancer-related mortality. Its incidence has increased rapidly in Western countries including Europe, North America and Australia [1, 2]. Despite improvements in perioperative treatments, the overall survival of patients throughout all tumor stages remains low with only 20% of patients surviving for more than 5 years [3–5].

In recent years, the inflammatory microenvironment has emerged as one of the focuses of cancer research and many publications have further supported the hypothesis of the immune system’s influence on cancer development and recurrence after cancer therapy, thus having a direct impact on disease-free and overall survival [6–8]. For EAC, a few studies with up to 130 cases of EAC showed a favourable outcome in tumors with high numbers of CD3- or CD8-positive T-cells [9–13].

In colon carcinoma, both the overall inflammation and the particular effect of special subtypes of tumor-infiltrating lymphocytes demonstrated that the number, type and location of tumor immune infiltrates have prognostic power and might be a superior way to classify those tumors over the AJCC/UICC TNM classification [14–19]. Recently, a publication described the relevance of a precise spatial subdivision of the invasion zone with respect to T cell distribution in colon carcinoma [20]. Nothing is known about the distribution and prognostic impact of T-cells in EAC.

MHC1 loss is a well-known escape mechanism of tumor cells to avoid T cell attack—there are no reliable findings about the frequency of MHC1 loss on the tumor cells of EACs.

For PD-L1, a well-defined immune checkpoint marker, conflicting data exist in EAC. This is among other things due to different analysis and especially scoring methods used in the past [21, 22]. The combined positive score (CPS) for PD-L1 was established for gastric and gastroesophageal junction adenocarcinomas serving as a predictive marker for PD1-inhibitor therapy [23]. Actually, clinical studies indicate a better progression-free and overall survival for PD1 treatment in tumors with PD-L1 positivity according to the CPS [24, 25].

Locally advanced EACs are particularly qualified for immune checkpoint blockade because of their limited (systemic) treatment options.

The aim of this work is to determine the extent and spatial distribution of T cell inflammation and its prognostic significance in a group of locally advanced (≥ pT2) primary resected EACs and to correlate these findings with possible tumor-resistance mechanisms against T cell attack.

Our hypothesis is that EACs are heterogeneously enriched with T-cells considering the tumor center and the tumor invasion zone. Furthermore, we assume that T-cell-rich EACs have a better prognosis in locally advanced tumor stages and that MHC class 1 loss is an important and in EAC underestimated tumor-escape mechanism.

We used a standardized software-based evaluation algorithm to measure T cell inflammation and its distribution within the tumor very precisely, objectively and reproducibly.

## Patients & methods

### Patients and tumor samples

Formalin-fixed and paraffin embedded tumor tissue of 99 patients with esophageal adenocarcinomas that underwent primary surgical resection therapy between 2013 and 2017 at the Department of General, Visceral and Cancer Surgery, University of Cologne, Germany was analyzed. The standard surgical procedure consisted of a transthoracic en-bloc esophagectomy with two-field lymphadenectomy (abdominal and mediastinal lymph nodes), reconstruction by formation of a gastric tube with intrathoracic esophagogastrostomy (Ivor-Lewis esophagectomy) [26]. Technical details of this operation are described elsewhere [27–29]. Follow-up data were available for all patients (Table 1). All procedures performed in this study involving human participants were in accordance with the 1964 Helsinki declaration and its later amendments or comparable ethical standards. The present study was approved by the University of Cologne Ethics Committee (reference number 20-1393) and written informed consent was obtained from all patients.

**Table 1:** Patients´ clinical and patho-anatomical tumor characteristics

		*n* = 99	%
Sex	Male	90	90.7
	Female	9	9.3
Age group	< 69	53	53.2
	> 69	46	46.8
Tumor stage	pT2	28	28.0
	pT3	67	68.0
	pT4	4	4.0
Lymph node metastasis	pN0	20	20.0
	pN +	79	80.0
Grading	G1	0	0
	G2	34	34.7
	G3	65	65.3

### Immunohistochemistry

Immunohistochemical stainings were performed on full tumor sections using the BOND MAX from Leica (Wetzlar, Germany) according to the protocol of the manufacturers. We used the following antibodies and protocols: CD3 (rabbit monoclonal, Thermo Fisher Scientific, Karlsruhe, Germany, citrate buffer 1:50), CD8 (mouse monoclonal, Dako Agilent, Waldbronn, Germany, citrate buffer 1:200), PD-L1 (rabbit monoclonal, clone 28–8 Abcam, Berlin, Germany, EDTA 1:100), MHC class 1 (and HLA A, HLA B) (rabbit monoclonal, Abcam, citrate 1:300).

### Software-based analysis of T cell infiltration

For the purpose of quantifying CD3- and CD8-positive T cells, slides were scanned using a NanoZoomer S360 (Hamamatsu Photonics, Herrsching, Germany) slide scanner. Subsequently, the analysis was performed using Visiopharm Analysis software (Munich, Germany). Tumor areas were defined as region-of-interest and divided into three parts: tumor center and tumor infiltration margin, further sub-divided into areas 50 µm above the infiltration margin (direction to the tumor center) and 300 µm beyond the infiltration border. All quantitative measurements were normalized to the total amount of counted cells.

### Strategy of evaluation of PD-L1 and MHC1

Two pathologists (A.Q. and H.L.) independently of each other scored PD-L1 and MHC class 1 manually. Scoring of PD-L1 followed the recommendations for gastroesophageal cancer. The CPS is defined as PD-L1-positive immune (including macrophages and lymphocytes) and tumor cells in proportion to all tumor cells and multiplied with 100. For MHC1, a homogenous and heterogeneous MHC1 expression on tumor cells was assessed as positive and homogenous loss of expression was counted as MHC1-negative (= MHC1 loss).

### Statistical analysis

Clinical data were collected prospectively according to a standardized protocol. SPSS Statistics for Mac (Version 21, SPSS) was used for statistical analysis. Interdependence between stainings and clinical data was calculated using the chi-squared and Fisher’s exact tests, and displayed by cross-tables. Group differences were calculated by the t-test or ANOVA, respectively. Univariate cox-regression analysis was performed for determination of interdependence between survival time and number of T-cells in the tumor. Survival curves were plotted using the Kaplan–Meier method and analyzed using the log-rank test. All tests were two-sided. *P* values < 0.05 were considered statistically significant.

## Results

### Patients' cohort

In total, 99 patients were fully analysable with a median age of 69 years (range 40–86 years). The patient cohort consisted of 90 men (90.9%) and 9 women (9.1%) (Table 1). Most adenocarcinomas analyzed here presented with a diffuse or solid tumor pattern and were classified as G3 (65.3%; Table 1). The median follow-up for the entire patients´ cohort was 40.7 months, observed death events were available from 85 patients (85.9%), 14 patients (14.1%) were still at life at the time of analysis and were censored. Only patients without neoadjuvant treatment prior to surgery were included in this analysis. The patients are stratified for preoperative therapy concepts according to pre-interventional diagnostic methods, which can lead to over- or understaging of the clinical tumor stage. Hence, in our patient cohort, tumors, which were understaged prior surgery, are included. Another aspect is that some patients are not suitable for neaodjuvant therapy due to comorbidity or aphagia. Furthermore, one tumor in our study revealed microsatellite instability and was excluded from statistical analysis.

### T cell infiltration

**Fig. 1:**
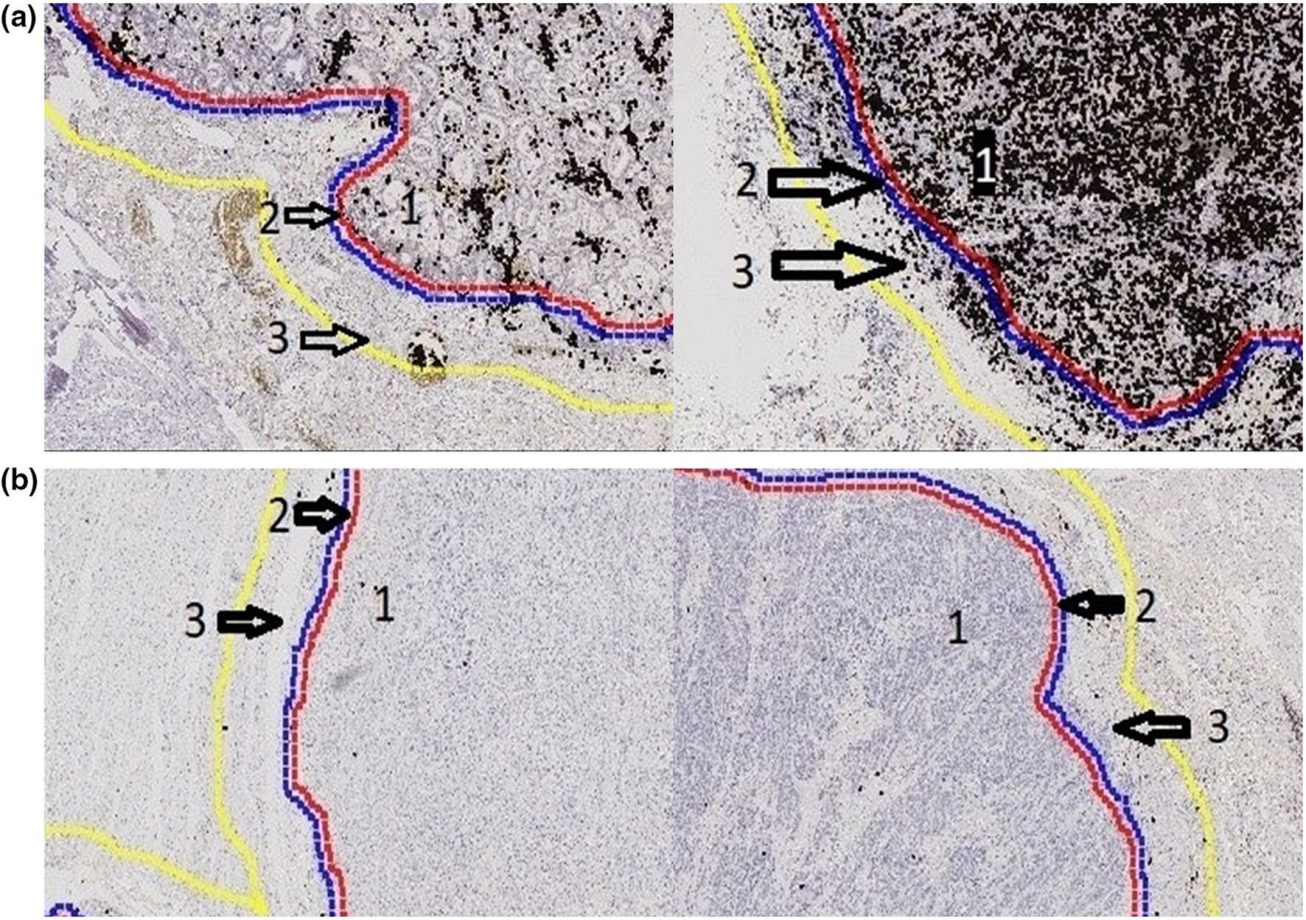
Density of T-cells considering different tumor areas. Variation of the total number of T-cells (**a**), the cumulative area of (**b**) and the density of T-cells (**c**) in the in outer and inner infiltration margin and tumor center

The total number of T-cells differed significantly between the tumor center and the outer and inner infiltration zone (Fig. 1a). For the tumor center, a median number of 29,800 T-cells were calculated (range 800–427,000), the outer invasive margin showed a median of 5700 cells (range 490–51,000) and the inner invasive margin a median number of 1260 T-cells (range 60–11,800) (*p* < 0.001). The cumulative area, as calculated by the total surface area covered by T-cells, showed similar results (Fig. 1b). The number of T-cells normalized to the tumor area (T-cells/mm^2^) showed no significant differences between the three areas within the tumor (Fig. 1c).

For dichotomous division of the continuous variable, the absolute T cell number was divided into a high and a low inflammatory group based on the upper quartile (75th percentile) (Table 2). There was a strong correlation of T cell richness between the outer and inner infiltration margins, as only four patients (4.0%) were considered CD3 high at the inner infiltration zone and CD3 low at the outer infiltration zone. The remaining patients were either low or high for CD3 in both infiltration zones (*p* < 0.001). The correlation between the infiltration zones and tumor center showed also a strong statistical correlation. However, within the group of CD3 high patients in the tumor center, only 55.0% (*n* = 8) and 66.0% (*n* = 13) of the tumors were also CD3 high at the infiltration zones (inner and outer, respectively) (*p* < 0.0001, respectively). In total, from patients with CD3 low in the tumor center 11 (11.1%) were considered CD3 high at the outer infiltration zone and eight patients (8.1%) at the inner infiltration zone.

**Table 2:** Correlation of T cell infiltration (CD3) with clinical characteristics

		CD3 outer infiltration zone					CD3 inner infiltration zone					CD3 tumor center				
		Low		High		*p* value	Low		High		*p* value	Low		High		*p* value
		*n*	%	*n*	%		*n*	%	*n*	%		*n*	%	*n*	%	
Sex	Male	68	75.6	22	24.4	1.000	67	74.4	23	25.6	0.448	69	76.7	21	23.3	0.684
	Female	7	77.8	2	22.2		8	88.9	1	11.1		6	66.7	3	33.3	
Age group	< 69	40	85.1	7	14.9	0.059	39	83	8	17	0.159	36	76.6	11	23.4	1.000
	> 69	35	67.3	17	32.7		36	69.2	16	30.8		39	75.0	13	25.0	
Tumor stage	pT2	21	77.8	6	22.2	0.563	19	70.4	8	29.6	0.490	36	70.4	11	29.6	0.671
	pT3	51	73.9	18	26.1		53	76.8	16	23.2		39	78.3	13	21.7	
	pT4	3	100.0	0	0		3	100	0	0		2	66.7	1	33.3	
Lymph node metastasis	pN0	15	75.0	5	25.0	1.000	14	70	6	30.0	0.562	12	60.0	8	40.0	0.082
	pN +	60	75.9	19	24.1		61	77.2	18	22.8		63	79.7	16	20.3	

A linear regression model was performed to assess a correlation between the number of CD3 and CD8 cells for each tumor area. A strong correlation between CD3 + and CD8 + cells could be seen in every single tumor area, the correlation coefficient (R) was 0.879 for the outer invasive margin, 0.857 for the inner invasive margin and 0.885 for the tumor center (*p* < 0.001, respectively).

To assess the influence of T cell infiltration with respect to survival time, univariate cox-regression analysis was performed. For the infiltration zone, a correlation between number of T-cells and overall survival (OS) could not be shown. For the tumor center, a correlation was present. However, the hazard ratio was small (hazard ratio 0.999 (0.9999–1.000, *p* = 0.023)) due to relatively small changes in the survival time in dependence of the increasing T cell number.

**Fig. 2:**
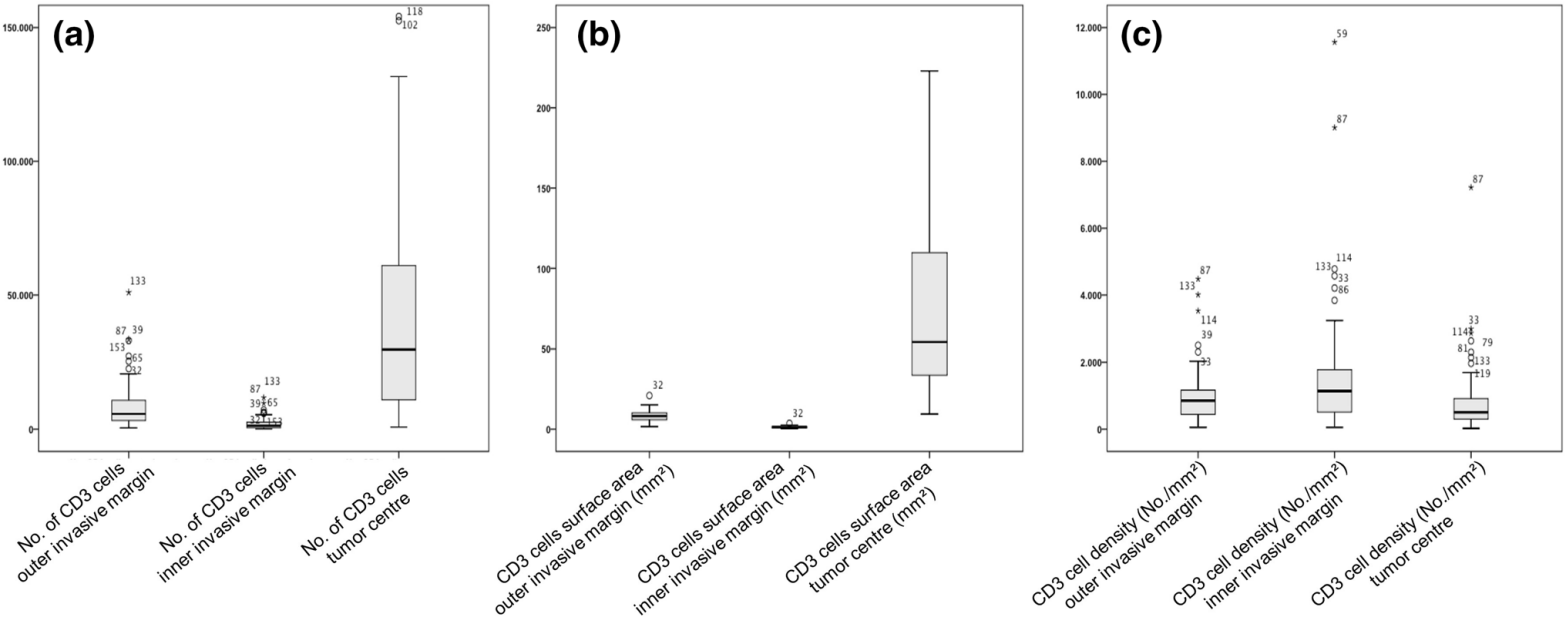
Overall-survival (Kaplan–Meier) depending on the T cell localization within the tumor. Compared overall survival of patients considering the T cell inflammation (high and low) at the outer invasive margin (**a**), inner invasive margin (**b**) and tumor center (**c**)

Subsequently, according to the upper quartile, dichotomous group comparisons were performed using Kaplan–Meier survival analysis. High numbers of T-cells in the infiltration zone, both outer and inner infiltration margin, did not affect OS (Fig. 2a and b). Considering the median survival after tumor resection, high amounts of T cell infiltration in the tumor center showed a significant prognostic effect of 41.4 months (95% confidence interval (95%CI) 22.1–60.1 months) compared to 16.3 months in T cell poor tumors (95%CI 8.7–22.4 months, *p* = 0.025). CD3 high tumors at any of the infiltration zones but not in the tumor center did not affect the OS (Fig. 2c) (*p* = 0.344). Therefore, only tumors with high CD3 expression in the tumor center are associated with a better OS, demonstrating that the overall T cell infiltration in the tumor center is prognostic in EAC. However, in multivariate analysis, CD3 expression fails to serve as an independent prognostic marker (Table 3).

**Table 3:** Multivariate cox- regression analysis for the impact of CD3 + cells in the tumor on overall survival (*HR *hazard ratio)

	Outer infiltration zone				Inner infiltration zone				tumor center			
	HR	95% confidence interval		*p* value	HR	95% confidence interval		*p* value	HR	95% confidence interval		*p* value
		Lower	Upper			Lower	Upper			Lower	upper	
Sex (male vs. female)	3.463	1.078	11.127	0.037	4.014	1.244	12.956	0.020	3.652	1.135	11.753	0.030
Age group (< 65 yrs. vs > 65 yrs)	1.330	0.795	2.225	0.277	1.338	0.806	2.221	0.260	1.246	0.756	2.054	0.389
pT (pT2 vs pT3/4)	1.507	1.017	2.234	0.041	1.455	0.982	2.155	0.061	1.457	0.976	2.174	0.065
pN_(pN0 vs pN +)	3.723	1.752	7.913	0.001	3.836	1.796	8.191	0.001	3.562	1.668	7.604	0.001
CD3 + (low vs high)	0.660	0.359	1.212	0.180	0.596	0.321	1.107	0.101	0.709	0.372	1.349	0.295

### PD-L1 expression and MHC class 1 (HLA A, HLA B)

To measure PD-L1 expression, the combined positivity score (CPS) was used and PD-L1 positivity was defined as a CPS > 1. 12 patients (21.2%) revealed as PD-L1 positive with a CPS of more than 1 (2–100) (Fig. 4a + b, Table 4). Loss of MHC class 1 expression on tumor cells was seen in 31 patients (32.0%, Fig. 4c + d, Table 4). A high PD-L1 expression was associated with advanced tumors (p = 0.009) in cross-table analyses. However, the calculated p values are statistically significant, but the results must be interpreted carefully due to the small number of 12 PD-L1-positive and 31 MHC1-negative cases. A correlation between MHC1 loss and clinical features could not be revealed (Table 4). Neither MHC class 1 loss nor PD-L1 expression was correlated with OS in Kaplan–Meier analysis (*p* = 0.123 and *p* = 0.232, respectively). A correlation between the number of CD3 + and CD8 + cells and MHC class 1 or PD-L1 expression was not detectable in cross-table analysis (data not shown).

**Table 4:** Correlation of expression of MHC1 and PD-L1 with clinical characteristics

		MHC1					PD-L1 (CPS)				
		Loss		High		*p* value	Negative		Positive		*p* value
		*n*	%	*n*	%		*n*	%	*n*	%	
Sex	Male	29	39.2	59	60.8	0.714	78	78.8	12	21.2	0.595
	Female	2	22.7	7	77.3		9	9.0	0	91.0	
Age group	< 69	18	39.1	28	60.9	0.192	45	95.7	2	4.3	0.030
	> 69	13	25.5	38	74.5		42	80.8	10	19.2	
Tumor stage	pT2	11	40.7	16	59.3	0.286	23	85.2	4	14.8	0.009
	pT3	20	29.9	47	70.1		63	91.3	6	8.7	
	pT4	0	0	3	100		1	33.3	2	66.7	
Lymph node metastasis	pN0	7	35.0	13	65.0	0.791	19	95.0	1	5.0	0.450
	pN +	24	31.2	53	68.8		68	86.1	11	13.9	

## Discussion

**Fig. 3:**
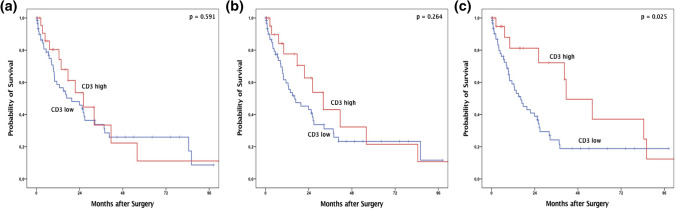
Computer-based evaluation considering the tumor center and the invasion zone (divided into a 50 μm wide inner zone and a 300 μm wide outer zone) with regard to their T cell content (magnification × 200). **a**: Two different tumors with high density of CD3 positive T-cells (black dots) separated in tumor center (1), tumor inner invasive margin (arrow 2: area between red and blue line; 50 µm) and outer invasive margin (arrow 3: area between blue and yellow line; 300 µm). **b** Two different tumors with low density of CD3 positive T-cells (just very few black dots) separated in tumor center (1) and tumor invasive margin (arrow 2: area between red and blue line; 50 µm) and outer invasive margin (arrow 3: area between blue and yellow line; 300 µm)

Our study takes into account a standardized, commercially available software-based evaluation algorithm considering large tumor sections. Image analyses offer a precise and reproducible measurement of T cell distribution in different parts of the tumor. It is well known that T cell infiltration correlates with outcome in EAC [14]. However, previous analyses of colon adenocarcinoma highlighted that especially the T cell-enriched invasion zone is prognostically relevant [17]. Berthel et al. has recently demonstrated the relevance of a further subdivision of the invasion zone for the colon carcinoma [20]. Our software-based application allowed a precise subdivision of the invasion zone into a 50 μm thin inner, tumor-facing zone and a 300 μm wide outer zone (Fig. 3). For EAC, this is the first study analyzing the impact of T cell infiltration of the different tumor parts.

We see significant differences in T cell enrichment not only between different tumors, but also within the same tumors. The software-based analysis revealed large differences in the T cell abundance with fluctuations from 800 to 427.000 T-cells in the tumor center or from 60 to 51.000 T cells in the invasion zone. Therefore, the invasion zone has fewer total numbers of T-cells than the tumor center. Within the invasion zone, the inner zone hardly shows any T cell infiltration with a four time lower average than in the outer zone (Fig. 3a and b). Anyway, we did not see any prognostic effect by considering the two different zones of the invasion zone separately from each other. But we can show a predictive relevance of T-cell-rich inflammation in EAC particularly driven by the T-cell-rich inflammation in the tumor center. This result is in line with the finding of a previous publication considering the tumor center of EAC using tissue micro-arrays [15].

The combination of a strong T-cell-enriched tumor center and a T-cell-enriched invasion zone does not increase the level of significance. T-cell-rich tumors are described in gastric carcinoma or colon carcinoma and are more commonly associated with DNA repair defects (e.g., microsatellite instability (MSI)) [30, 31]. However, esophageal adenocarcinomas do not fit in the four defined TCGA subgroups of gastric adenocarcinomas [32, 33]. So, the MSI-subtype is, in contrast to gastric adenocarcinomas, very rare in EAC. In our study cohort, we found microsatellite unstable EACs in only 0.6%, which was published previously [34]. This conforms to the TCGA data, where no MSI subtype was found in their cohort [32]. We were able to exclude a single MSI case from our (highly T-cell-rich) EACs.

Additionally, we considered two possible tumor-escape mechanisms: protein expression of PD-L1 on the tumor cells and corresponding inflammatory cells (CPS) as well as down-regulation of the neo-antigen presentation proteins of the MHC class 1 complex.

**Fig. 4:**
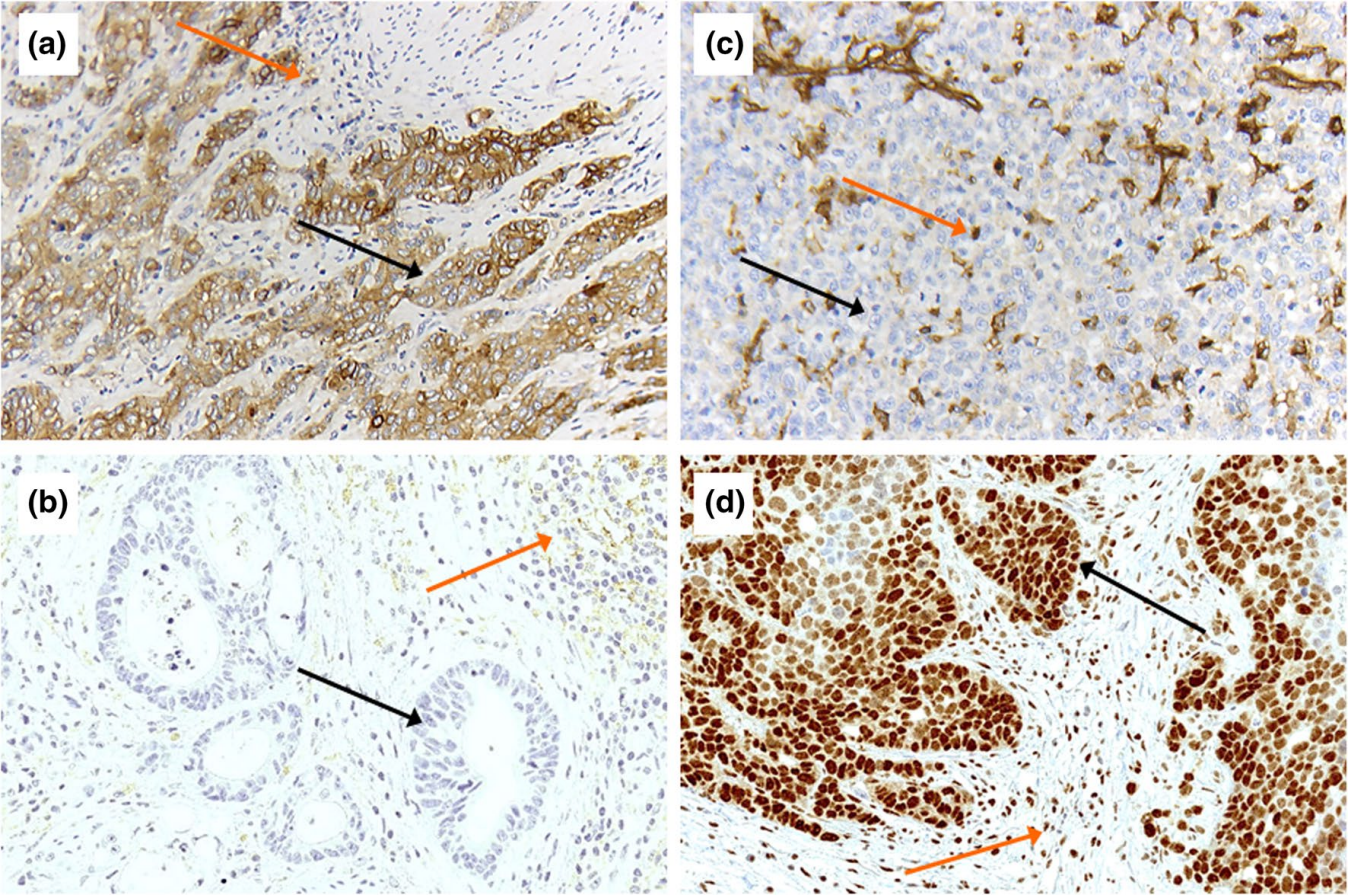
Immunohistochemical detection of PD-L1 and MHC1. A + B: PD-L1 strongly positive (**a**) and with low expression (**b**) using the Combined Positive Score (CPS 100) (magnification × 200). Black arrows show PD-L1 tumor cells positive (**a**) and negative (**b**). Orange arrows show PD-L1 positive inflammatory cells. C + D: MHC1 loss (**c**) and preserved expression (**d**) (magnification × 200). Black arrows show tumor cells with complete loss of MHC1 protein (**c**) and preserved nuclear staining (**d**). Orange arrows show internal positive control of MHC1 positive inflammatory cells

The effectiveness of a drug blockade of the PD-L1 / PD-1 axis has been impressively demonstrated in recent years in non-small cell lung carcinomas and malignant melanomas (among others) [35–37]. For EAC, first results of clinical studies are promising regarding a better prognosis for PD-1 therapies in PD-L1-positive tumors evaluated by CPS [24, 25]. The data on the extent and prognostic significance of PD-L1 expression in the EAC vary in the literature and ranges from 2.9% to 40% [38, 39]. These are the reasons for these differences of primary antibodies used against PD-L1 (we have used the FDA-approved clone 28-8) or the underlying evaluation criteria (Tumor proportion score (TPS) or combined consideration of carcinoma cells and inflammatory cells (CPS), which we applied, Fig. 4a).

Due to their mutational spectrum, tumors have many foreign proteins that present to the immune system via the MHC class 1 complex on the tumor cell surface. It suggests that down-regulation of MHC class 1 may be an effective mechanism of tumor cells to evade detection by the immune system.

In fact, some studies have presented this mechanism as highly relevant or as a reason for lack of response rates for immune checkpoint inhibitor treatment [40, 41].

There are no reliable data on the frequency of MHC class 1 downregulation in EAC.

In our tumor cohort, we detected a MHC1 loss in 32% of the cases (Fig. 4b). Compared with other tumor entities, such as malignant melanoma, which shows an MHC1 downregulation in up to 45% of the cases, a comparable frequency can be found in EAC [42]. So, MHC class 1 loss could be a relevant tumor immune escape mechanism in EAC.

Possible limitations of our study are the retrospective character of the investigation and the sole analysis of surgical specimens. It would be interesting to determine the T cell content of primary endoscopic tumor biopsies to determine the predictive power of T cell inflammation prospectively, also in settings with applied immune checkpoint therapies.

Since our study has shown that the T-cell-rich tumor center is significantly prognostic and that well-obtained biopsy material can reach this tumor region, we can speculate that endoscopic material also produces comparable results.

Future clinical trials investigating the efficacy of checkpoint inhibitors in EAC will show how much the T cell-rich subtype of EACs and MHC1 tumor cell loss as a tumor-escape mechanism influence clinical response to therapy.
